# A Rare Presentation of an Invasive Ductal Carcinoma of Ectopic Axillary Breast Tissue

**DOI:** 10.7759/cureus.9928

**Published:** 2020-08-21

**Authors:** Shobha Mandal, Mary Grace Bethala, Chandrakala Dadeboyina, Sushmita Khadka, Vineela Kasireddy

**Affiliations:** 1 Internal Medicine, Guthrie Robert Packer Hospital, Sayre, USA; 2 Internal Medicine, GlobeHealer, Philadelphia, USA; 3 Hematology and Oncology, Guthrie Robert Packer Hospital, Sayre, USA

**Keywords:** ectopic breast tissue, invasive ductal carcinoma

## Abstract

Ectopic breast tissue (EBT) is a rare entity and can present anywhere along the milk line, including the axilla, inframammary region, thighs, perineum, groin, and vulva. However, the axilla is the most common area of presentation. EBT can present as supernumerary breasts or aberrant breast tissue. Malignancy arising in EBT is rare, but the most common morphological variant is invasive ductal carcinoma. We report a case of a 43-year-old woman, a smoker with a family history of breast cancer, who presented to our clinic with a small mass in the right axillary area. After monitoring it for one year, the mass increased in size, so she returned to the clinic and decided with her care team to excise the mass. Histopathology showed invasive mammary adenocarcinoma arising in EBT and was diagnosed as right accessory stage I breast cancer. This case illustrates the imperative that any mass in the axillary region should be thoroughly assessed to rule out carcinoma in the accessory axillary tissue for timely management.

## Introduction

Breast cancer is one of the most common cancers in women, among which 0.3% to 0.6% of cancer cases occur in ectopic breast tissue (EBT). EBT is a rare entity that can be present anywhere along the milk line, but most commonly in the axilla. It can present as supernumerary breasts or aberrant breast tissue [1]. Supernumerary breast development is hormone dependent, like healthy breast, that begins to evolve during pregnancy or puberty and has a similar risk of undergoing benign and malignant transformations [2,3]. It may contain glandular tissue only or can be in combination with the nipple-areola complex. The aberrant breast tissue contains disorganized secretory glandular tissue [4]. Accessory breast tissue may also be divided into polymastia (breast tissue composed of the ductal system connecting to overlying skin) and polythelia (accessory nipple or areola) [5]. We report a case of a 43-year-old woman who presented to our clinic with a small mass in the right axillary area that was histopathologically diagnosed as invasive mammary adenocarcinoma arising in EBT. 

## Case presentation

A 43-year-old premenopausal woman with a past medical history of early-stage melanoma of the right thigh, treated with wide excision, was found to have a small mass in the right axillary area that was non-tender, rubbery, and firm. She reported no other concerns. She was an active smoker, and her mother was diagnosed with breast cancer at age 55 years. On the physical exam, both breasts and left axilla were healthy, but in the right axilla, a soft, mobile, non-tender mass measuring 2 cm x 2 cm was palpated. There were no extramammary nipples along the milk line. No other findings from the physical examination were remarkable. 

**Figure 1:**
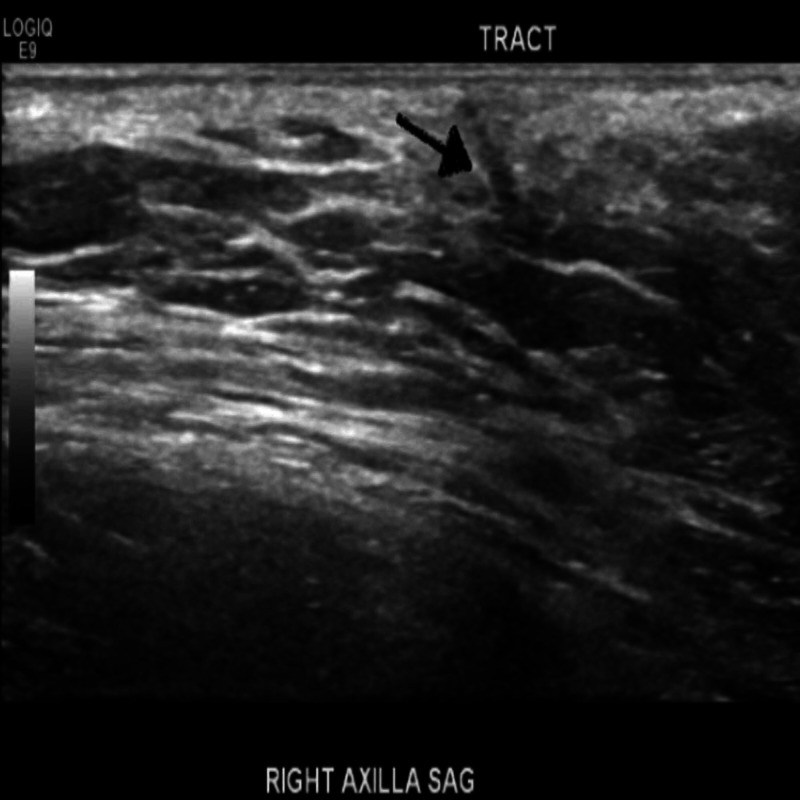
Right axillary ultrasound showing a vague hypoechoic area with an apparent tract extending through the skin (black arrow pointing the tract).

A right axillary ultrasound showed a vague hypoechoic area within her right axillary skin with an apparent tract extending through the skin that was suspected to be epidermal inclusion, possibly a sebaceous cyst (Figure 1).

**Figure 2:**
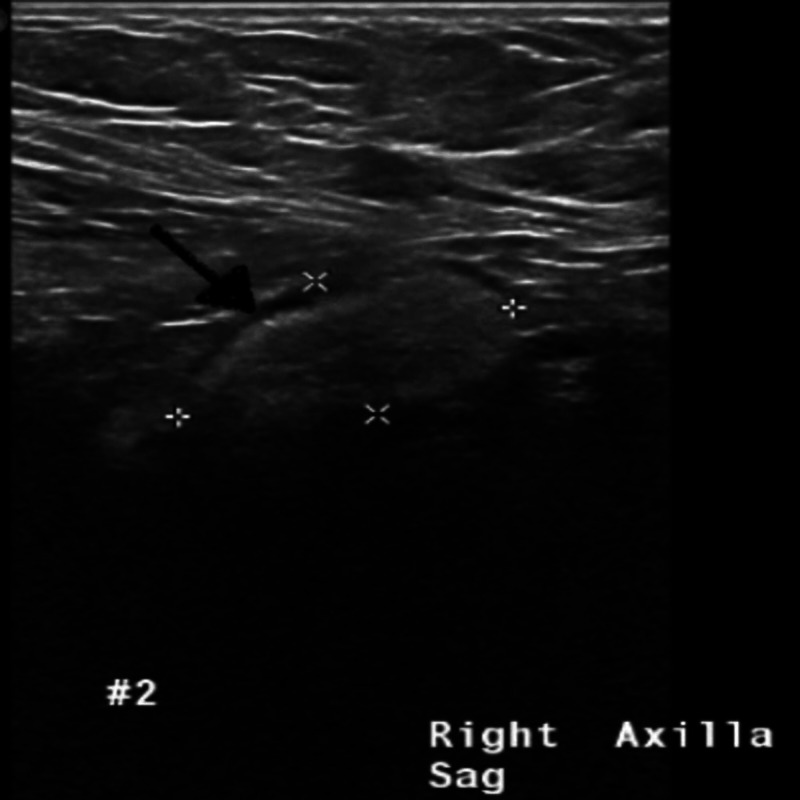
Repeat ultrasound of right axilla showing asymmetric focal density (black arrow pointing the focal density).

The findings of her mammogram were negative bilaterally without evidence of malignancy. The patient decided to observe the mass and later came back for a follow-up visit in one year. The repeat physical exam revealed that the mass had grown and now measured 3 cm x 3 cm. It was non-tender and firm. Repeat ultrasound of the bilateral breast showed fibroglandular density and asymmetric focal density in her right axilla (Figure 2).

**Figure 3:**
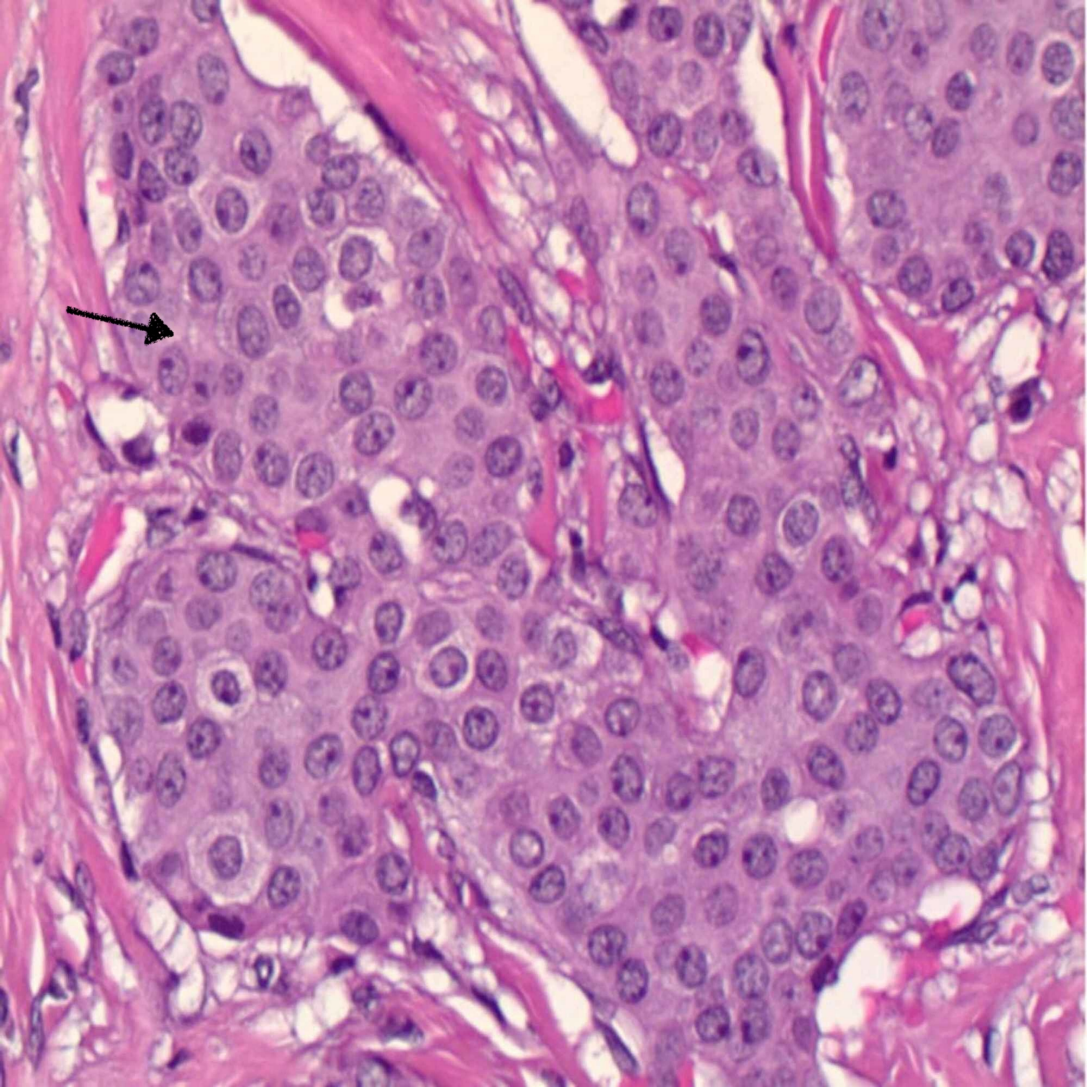
Moderately differentiated invasive ductal carcinoma (shown by black arrow). No tubule formation and intermediate grade nuclei.

**Figure 4:**
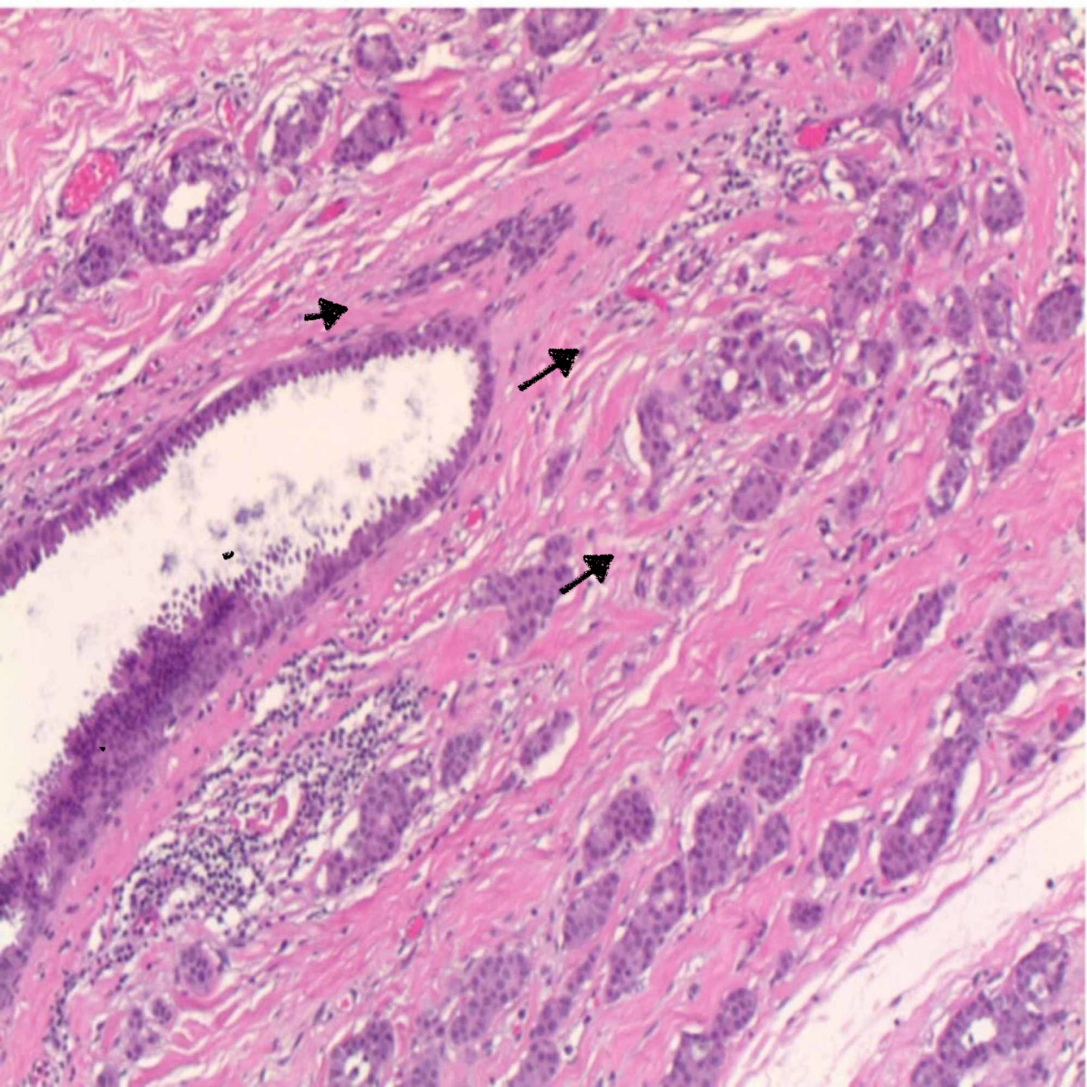
Carcinoma arising from breast ducts. Black arrows showing tumor cells.

As the size was increasing, the patient and her care team decided to excise the mass. On excision, the mass was deep rooted, measuring 3 cm x 3 cm. Histopathology showed an invasive mammary adenocarcinoma, arising in EBT and involving the dermis and subcutaneous fat. Immunohistochemistry revealed the mass was estrogen receptor 94%, progesterone receptor 94%, and human epidermal growth factor receptor 2 (HER2) negative and Ki-67 test was 26% with negative margins (Figures 3, 4). 

 

The biopsy was negative for the lymph node involvement. Therefore, the patient was diagnosed with right accessory stage I breast cancer (tumor: 1; node: 0; metastasis: 0). 

Given cancer’s early stage, and that it was node negative, hormone receptor positive, and HER2 negative, an Oncotype Dx® test (Genomic Health, Inc., Redwood City, CA) was performed, which showed a low score (6), indicating no benefit with adjuvant chemotherapy. She was started on adjuvant endocrine therapy with oral tamoxifen 20 mg per day after the completion of adjuvant axillary radiation therapy. 

## Discussion

Accessory breast tissue arises from persistent milk lines that are thickened strips of ectoderm running along the ventral surface of the body from the anterior axillary fold to the medial aspect of inguinal fold bilaterally, which normally involute during embryogenesis except in the thoracic region where they give rise to breast tissue [1,6]. EBT can become apparent during puberty and pregnancy because of hormonal influences and may contain either glandular tissue only or may contain a combination of glandular tissue and the nipple-areola complex [6]. EBT is usually located in axilla (60% to 70% of cases) but can also be present in the inframammary region (5% to 10% of cases) and rarely in thighs, perineum, groin, and vulva [7]. 

EBT is present in both sexes but more common in women, with an estimated incidence of 0.22% to 6% [8]. The incidence of malignancy arising in EBT is 0.3% to 0.6% [8]. Invasive ductal carcinoma is the most common histological type, accounting for 79% of all ectopic breast cancer, followed by lobular, medullary, mucinous, apocrine, papillary, and cystosarcoma phyllodes [9]. Invasive ductal carcinoma has five subtypes: mixed (45.6%), classical (30.4%), tubulo-lobular (13.5%), solid (6.4%), and alveolar (4.1%), among which tubulo-lobular has the best survival and prognosis [10]. 

Patients are usually unaware of the presence of EBT and often ignore it as pigmentation or fat tissues unless it becomes pathologically involved in the form of inflammation, benign lumps, and malignancy. Treatment is usually delayed due to a wide variety of differentials (lymphadenopathy, lipoma, abscess, seroma, hidradenitis, fibroadenoma, and cutaneous lesions) and a lack of awareness regarding accessory breast cancer [11]. Patients with EBT may present with a mass in the axilla, around or under the breast, or anywhere along the milk line. The mass may be dark brown and is usually non-tender. Less commonly, it may present with edema, breast pain, non-specific discomfort, or extramammary nipples along the milk line. In multiple case reports, the most common tumor reported in EBT was fibroadenoma, and, less commonly, phyllodes tumor and mammary carcinoma [12]. 

As it is a rare presentation and the location is extramammary, to diagnose ectopic breast carcinoma accurately, one should focus on a detailed history taking, physical examination, imaging, and tissue sampling, such as fine-needle aspiration and core-needle biopsy [13]. MRI can assess the extent of the tumor for diagnosis and staging in patients presenting with a mass in the milk line [14]. 

Treatment is individualized based on the stage and location of the EBT, but the first step is generally excision of the mass with clear margins. The literature offers no formal guidance on adjuvant radiotherapy nor its dose, fractionation, and treatment [15]. If there is involvement of both healthy tissue and EBTs, which can rarely occur, treatment is conventionally either a lumpectomy or mastectomy with sentinel lymph node biopsy. Mastectomy is not indicated if clinical examination, mammography, and ultrasonography of the anatomic breast reveal no signs of disease. 

Patients in whom mastectomy is not performed should be monitored via regular follow-up with clinical examinations and breast imaging to exclude any later manifestations of a primary occult neoplasm. The accurate prognosis of the malignancy of EBT is unknown because of limited data in the literature. In a retrospective series, carcinoma of axillary EBT was found to have a worse outcome compared to anatomic breast cancer as it is associated with early metastasis to the axillary lymph nodes [7]. 

## Conclusions

This case report describes a premenopausal woman who presented with a small axillary mass that was excised after one year of monitoring and growth. The mass was histopathologically diagnosed as invasive mammary adenocarcinoma arising in EBT. Prophylactic excision of EBT is also recommended where indicated. Any mass in the milk line axillary region must be thoroughly assessed to rule out carcinoma and optimize patient outcomes. 
